# Repair of a thoracic incisional hernia in a patient after elective abdominothoracic esophageal resection

**DOI:** 10.1186/s13019-026-04661-1

**Published:** 2026-08-10

**Authors:** Felix Rühlmann, Andrew McIntyre, Jochen Gaedcke

**Affiliations:** 1 Department of General and Visceral Surgery, Städtisches Klinikum Karlsruhe, Moltkestr 90, Karlsruhe, 76133 Germany; 2 Institute of Diagnostic and Interventional Radiology, Städtisches Klinikum Karlsruhe, Karlsruhe, Germany

**Keywords:** Thoracic incisional hernia, Thoracic hernia repair, Hernia, Case report, Esophageal resection

## Abstract

**Background:**

Herein, we report from a 70-year-old male patient being diagnosed with a thoracic intercostal incisional hernia who prior underwent abdominothoracic surgery due to carcinoma of esophagogastric junction. These hernias are very rare, therefore there exist no established techniques for surgical treatment.

**Case presentation:**

The patient has described a pain in the right-sided hemithorax and a bulge while coughing for several weeks. He had a long history of smoking. Clinical examination revealed an intercostal anterolateral hernia which was confirmed via radiological computed tomography (CT) scan with a defect size of 7.5 cm.

**Conclusions:**

Subfascial hernia mesh repair is feasible in thoracic incisional hernia and was well-tolerated by the patient. However, these hernias remain very rare therefore there are no common recommendations for standardized treatment. Future data should be collected centrally using registered data bank.

**Supplementary Information:**

The online version contains supplementary material available at 10.1186/s13019-026-04661-1.

## Background

Thoracic incisional hernias are very rare phenomena. The first pulmonary hernia was described in 1499 [1]. A classification of Morel-Lavallee is established respecting anatomic location (cervical, thoracic, diaphragmatic) as well as aetiology (congenital or acquired) [2]. The majority of lung hernias (approximately 80%) are acquired hernias including spontaneous, pathological and traumatic genesis [3]. Due to the lack of published data, there is no common guideline for treating thoracic intercostal hernias.

## Case presentation

Herein, we report of a 70-year-old male patient who prior underwent hybrid abdominothoracic esophageal resection after neoadjuvant chemotherapy due to an adenocarcinoma of the esophagogastric junction type I (Siewert classification EGJ I; ypT0, ypN0(0/34), L0, V0, Pn0, G3, R0) at the Department of General and Visceral Surgery at Städtisches Krankenhaus Karlsruhe in December 2024.

During follow-up, he presented as an out-patient at our department with pain located in the right-sided hemithorax and a thoracic bulge ibidem while coughing. The patient has had a long-term history of chronic nicotine abuse (cumulatively 120 packyears).

**Fig. 1:**
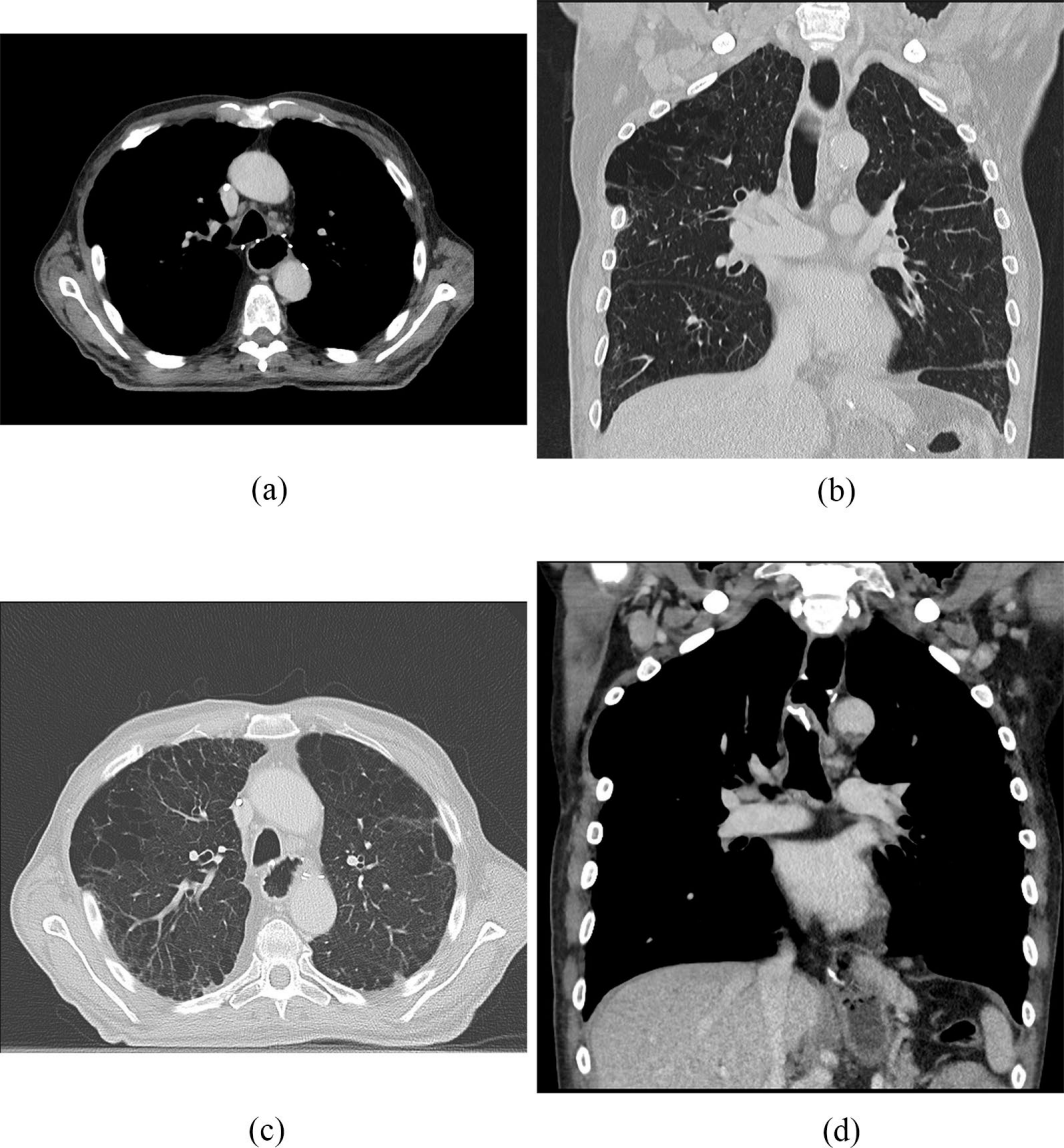
**A-D** Axial and coronal CT scans in soft tissue as well as in lung window showing a significant distance between third and fourth rib of the right hemithorax

In the physical exam, we saw an intercostal incisional thoracic hernia after thoracotomy. Computed tomography (CT) revealed an anterolateral intercostal lung hernia (size: 7.5 cm) between third and fourth rib with distinct thinning of the right thoracic wall (see Fig. 1A-D).

Due to the worsening clinical symptoms of the patient we saw an indication for surgical treatment.

Thoracic scar was reopened followed by preparation of pectoral fascia till the centre of the hernia. The hernia sac respectively scarred musculature was then opened. We then saw a significant distance (approximately 7 till 8 cm) between third and fourth rib anterolateral without any adhesions of the lung. Pectoral muscle was then further mobilized and third and fourth rib were then reapproximated using a total of five stiches of polydioxanone suture 1.0 (PDS 1.0). We then placed a 25 × 20 cm mesh below the pectoral muscle fixing it with prolene 2.0 suture ensuring no relevant contact between the mesh and the lung. A redon drain was placed. The pectoral muscle was then reclosed under enhanced positive end-expiratory pressure (PEEP).

The patient made an uneventful recovery and did not have any postoperative complications concerning hernia treatment.

During the following surveillance, the patient presented without any pain medication and better general condition.

The patient presented as an out-patient two weeks after discharge from hospital. He was in a very good condition and did not need any pain medication anymore.

Clinical and radiological follow-up was performed in January 2026. Clinical examination as well as radiological slides neither showed hernia nor tumor relapse.

## Discussion and conclusions

Predisposed factors regarding the development of lung hernias have been discussed in the past. Common risk factors are associated with increased intrathoracic pressure such as obesity, chronic obstructive pulmonary disease (COPD) or increased coughing due to smoking [4]. Further risk factors comprehend tissue weakness, impaired wound healing, malnutrition, steroid therapy, diabetes and other comorbidities.

Our patient showed two risk factors such as chronic nicotine abuse as well as beginning signs of malnutrition (BMI 19,4 kg/m^2^; height: 176 cm, weight: 60 kg). He previously underwent prehabilitative procedures at our ward including intravenous parenteral nutrition and breathing therapy.

Although the patient made an uneventful recovery, it still represents a single case in a single centre.

Postoperatively, an anatomical weakness of the intercostal muscles in this patient is anticipated. An incomplete distribution of the three intercostal muscles (internal, medial and external intercostal muscles) within the intercostal space are associated with weak sites of the thoracic wall [5].

As sufficient long-term data is not available due to too few published cases, both conservative and surgical treatments are feasible. Asymptomatic patients do not necessarily need surgical therapy. However, increasing size of the hernia, pain, or signs of impending incarceration represent indications for surgical treatment.

The usage of autologous tissue whenever possible was initially recommended for the repair of the defect [6]. Synthetic materials like Dacron, Ivalon, Teflon, GoreTex or Marlex are also acceptable if local tissues are not available [7]. Techniques like direct suturing, mesh repair, complex muscle flaps or the usage of laminar hooks have been described previously [8–10].

Minimally invasive thoracoscopic approaches were also reported [11, 12].

To evaluate the long-term outcome after different (surgical) treatments of thoracic incisional hernias a larger number of case reports and multicenter analysis are needed.

Subfascial mesh repair is feasible and a safe procedure in thoracic incisional hernia and was well-tolerated by the patient. The patient reported from a significant improved quality of life postoperatively.

It is important to state that the occurrence of a thoracic incisional hernia in this case might have been avoided by an initial minimally invasive approach regarding esophageal resection, which is nowadays our standard of treatment.

## Supplementary Information

Below is the link to the electronic supplementary material.

Supplementary Material 1

Supplementary Material 2

Supplementary Material 3

Supplementary Material 4

Supplementary Material 5

Supplementary Material 6

Supplementary Material 7

Supplementary Material 8

Supplementary Material 9

Supplementary Material 10

Supplementary Material 11

Supplementary Material 12

## Data Availability

The datasets used and/or analysed during the current study are available from the corresponding author on reasonable request.

## Abbreviations

CT: Computed tomography
EGJ: Esophagogastric junction
PDS: Polydioxanone suture
PEEP: Positive end-expiratory pressure
PVDF: Polyvinylidenfluorid
